# Diverse Functions of Plant Zinc-Induced Facilitator-like Transporter for Their Emerging Roles in Crop Trait Enhancement

**DOI:** 10.3390/plants11010102

**Published:** 2021-12-30

**Authors:** Varsha Meena, Shivani Sharma, Gazaldeep Kaur, Bhupinder Singh, Ajay Kumar Pandey

**Affiliations:** 1 Department of Biotechnology, National Agri-Food Biotechnology Institute, Sector 81, Sahibzada Ajit Singh Nagar 140306, India; varsha.meena@nabi.res.in (V.M.); shivanisharma.mtech2012@gmail.com (S.S.); gazaldeep.gk@gmail.com (G.K.); 2 Regional Centre for Biotechnology, Faridabad 121001, India; 3 Centre for Environment Science and Climate Resilient Agriculture, ICAR-IARI, New Delhi 110002, India; bhupindersinghiari@yahoo.com

**Keywords:** iron, zinc, antiporters, phytosiderophores, transport, iron deficiency, micronutrients

## Abstract

The major facilitator superfamily (MFS) is a large and diverse group of secondary transporters found across all kingdoms of life. Zinc-induced facilitator-like (ZIFL) transporters are the MFS family members that function as exporters driven by the antiporter-dependent processes. The presence of multiple ZIFL transporters was shown in various plant species, as well as in bryophytes. However, only a few ZIFLs have been functionally characterized in plants, and their localization has been suggested to be either on tonoplast or at the plasma membrane. A subset of the plant ZIFLs were eventually characterized as transporters due to their specialized role in phytosiderophores efflux and auxin homeostasis, and they were also proven to impart tolerance to micronutrient deficiency. The emerging functions of ZIFL proteins highlight their role in addressing important traits in crop species. This review aims to provide insight into and discuss the importance of plant ZIFL in various tissue-specific functions. Furthermore, a spotlight is placed on their role in mobilizing essential micronutrients, including iron and zinc, from the rhizosphere to support plant survival. In conclusion, in this paper, we discuss the functional redundancy of ZIFL transporters to understand their roles in developing specific traits in crop.

## 1. Introduction

The major facilitator superfamily is one of the second largest membrane transporters that is ubiquitously present in living organisms. The members of this family are composed of secondary active transporters that can utilize the electrochemical gradients to usher the transport of the diverse range of substrates [1,2]. The nature of the MFS family led to their classification into three groups, namely uniporters, symporter and antiporters transporters [3]. Amongst the plant MFS gene families, some are well studied for their transport function of substrates, including sugar, nitrate, phosphate transporters and ferroportins [4,5,6,7]. These classes of transporters also performs various functions, including selective transport of the substrates across the plasma membrane and regulated processes such as polar auxin transport and xenobiotic sequestration [8]. Plants are subjected to different nutrient stresses that include macro- and micro-nutrient stresses. Multiple reviews have focused on plant adaption to macronutrient stress conditions [9,10,11]. Micronutrient stress, including excess and deficiency, resulted in changes in the different metabolic parameters, including the oxidative stress and reactive oxygen species [12]. At the genetic level, the influence on the global gene expression patterns were also noted. Such an effect could influence the specific transporters, metal chelators and genes involved in the uptake and redistribution of metals in a tissue-specific manner. Iron (Fe) and Zinc (Zn) are important micronutrients that impact the plant physiology, growth and productivity. Multiple plant proteins of MFS gene families were identified for their wide array of roles. In addition, the new members of plant MFS proteins, such as ferroportin and zinc-induced facilitator-like (ZIFL) proteins, are now being explored for their role in metal homeostasis, especially in regulating the flux of two important micronutrients, viz Fe and Zn.

Micronutrients, including Fe and Zn, in grains are required for germination, seedling development and provide primary nutritional sources for humans, especially for people consuming cereals as staple foods [13]. However, both deficient and excess Zn impair the physiological and biochemical processes of the plant [14,15]. Zn deficiency causes yield loss and reduces crop quality [16,17], while Fe deficiency could impair several essential functions, including photosynthesis, respiration and redox reactions [18]. Therefore, plants must recruit the mechanisms that could entail the necessary uptake and mobilization of Fe and Zn forms from the rhizosphere to the plant tissue. One such gene family encoded by ZIFL genes is emerging as a significant player to assist in mobilizing rhizospheric Zn^2+^ in the soluble form [19]. In addition to this, ZIFL-encoded transporters are able to mobilize Fe by secreting phytosiderophores (PS) in the rhizospheric region. ZIFL transporters belong to the category of antiporters that require energy derived from the concentration gradients [3]. The current review highlights the recent advances that suggest an essential role of ZIFL in different biological functions and substrate transport. This information will provide new avenues for the genetic manipulations of ZIFL genes, using biotechnological approaches to develop unique traits.

## 2. ZIFL in Plants: EXPANDING Members, Yet Evolutionarily Conserved

**Figure 1:**
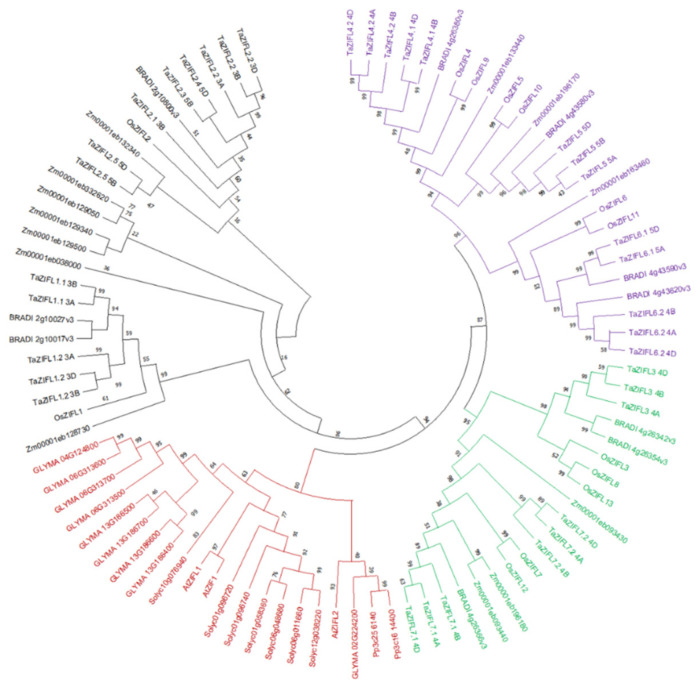
Phylogenetic analysis of ZIFL protein sequences. The putative ZIFL sequences for *Zea mays* (13 members), *Brachypodium distachyon* (10 members), *Glycine max* (9 members), *Solanum lycopersicum* (7 members) and *Physcomitrella patens* (2 members) Pfam ID PF07690 were used to extract MFS sequences from Ensembl, followed tree construction along with the rice (13 members), wheat (35 members) and *Arabidopsis* (3 members) ZIFL protein sequences.

Members of the *ZIFL* family are widely distributed among archaea, eubacteria, fungi and plants, including monocots, dicots, gymnosperms, ferns and mosses. The first plant *ZIFL* genes were identified in *Arabidopsis thaliana* and are referred to as *AtZIF1*, *AtZIFL1* and *AtZIFL2*, respectively [19]. A comparative analysis of the *ZIFL genes* from multiple plant species, including rice (*Oryza sativa*), wheat and maize, was performed that resulted in the unique features of the *ZIFL* proteins (Table 1). Phylogenetic analysis of multiple ZIFL proteins suggest a unique distribution pattern that is attributed to their closeness (Figure 1). On the basis of phylogeny distribution, we can observe that all the dicot ZIFL proteins fall in the same clade (red colored). This particular clade also includes members from mosses, i.e., *Physcomitrella patens (Pp3c256140* and *Pp3c1614400)*. Interestingly, ZIFL proteins from mosses show highest closeness with AtZIFL2. The expansion of the ZIFL protein members in other plant species, such as rice, maize, wheat and *Brachypodium* with the large numbers, suggests their importance in monocot species.

**Figure 2:**
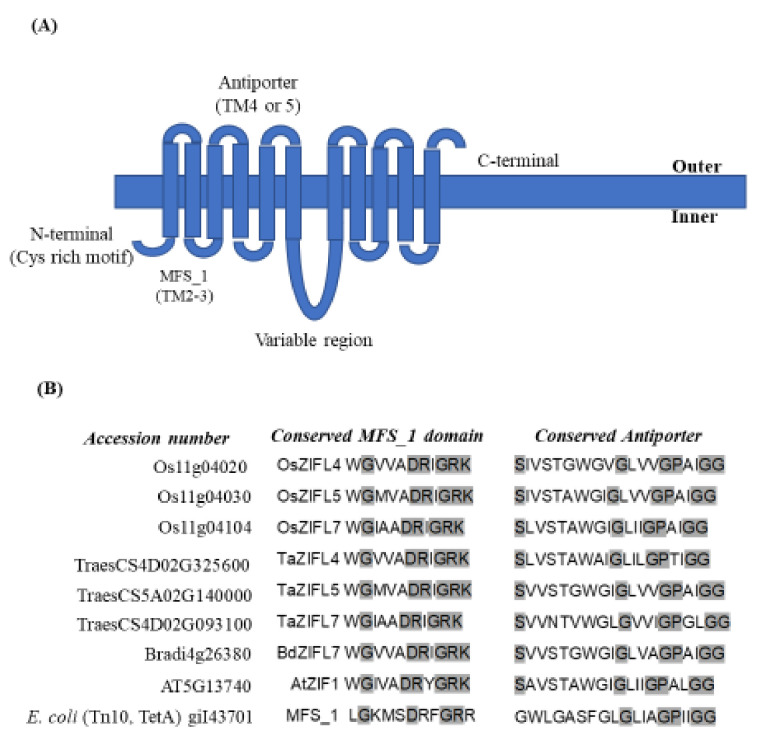
ZIFL family sequence signatures. (**A**) Schematic representation of the ZIFL bearing the MFS_1 signature (cytoplasmic loop between TM2 and TM3: MFS signature) and antiporter signature (TM4/5). (**B**) ZIFL-specific conserved-residue signature domains for MFS and antiporter sequences in rice (OsTOM1-ZIFL4, OsTOM2-ZIFL5 and OsTOM3-ZIFL7); wheat (TaTOM1-ZIFL4.1/4.2, TaTOM2-ZIFL5 and TaTOM3-ZIFL7.1/7.2), *Brachypodium* (*BdTOM1.1*- Bradi4g26380), *Arabidopsis* (AtZIF1) and *E. coli* (MFS_1). The conserved residue for MFS: G-x(3)-D-[RK]-x-G-R-[RK] residues are highlighted, and the Antiporter S-x(8)-G-x(3)-G-P-x(2)-G-G residues are highlighted.

At the structural level, a typical ZIFL protein structure consists of 11–13 transmembrane (TM) domains typically consisting of MFS_1 (Pfam ID:PF07690) and an antiporter domain with a signature sequence. The MFS_1 region usually spans in the second/third TM domain (Figure 2A). The antiporter-signature-conserved sequences are present on either the fourth or fifth TM domain of ZIFL protein. Alignment of ZIFLs of different plant species (rice, wheat, *Brachypodium* and *Arabidopsis*) ZIFL suggested highly conserved residues for either the MFS_1G-x(3)-D-[RK]-x-G-R-[RK] or antiporter domain S-x(8)-G-x(3)-G-P-x(2)-G-G sequences, as represented in Figure 2B [20]. This structural alignment of multiple proteins has suggested high similarity and conservation of the domains within a subset of ZIFL proteins, thereby categorizing them as a distinct family of transporters within the MFS gene family. In terms of unique features, one could signify the absence of conserved tryptophan (W) in the *E. coli* MFS_1 compared to the MFS domain of plant ZIFL (Figure 2B). Additionally, multiple plant ZIFL genes were identified, and their number varied based on the plant species. For example, thirteen *ZIFL* genes were reported in rice, ten in both *Zea mays* and *Brachypodium* and 35 genes in the hexaploid wheat (including all homoeologs) [21,22].

### 2.1. Overlapping Transcriptional Response of ZIFL

Under Fe-deficiency, ionomic changes are reported in plants tissues [23]. This broad specificity of metal uptake could be accounted by the surge in the expression of the broad metal acquisition system (strategy I or strategy II components) that are regulated during the Fe-deficiency, including ZIF/ZIFL genes [23,24]. Earlier, AtZIF1 was characterized as a vacuolar membrane MFS protein that provides tolerance to Zn excess and enhanced vacuolar sequestration of nicotianamine (NA), yet causes changes in Fe-homeostasis [19,25]. Loss-of-function and overexpression studies have demonstrated that ZIF1 expression is critical for both Fe and Zn homeostasis [25]. These evidences provided the preliminary clue that ZIF genes play an important role in the Fe and Zn partitioning at the tissue level. It was observed that most of the ZIFL genes, either from dicots or monocots, are differentially expressed during the changing Fe and Zn regimes [19,26]. The recent studies in *Arabidopsis*, using the pZIFL-GUS reporter system, confirms that the ZIFL expression is also dependent on the doses of Fe and Zn [27]. The *Arabidopsis zif1/zifl1* mutants show an increase in Zn and Fe sensitivity by enhancing Zn concentrations in the shoot that was linked to the higher shoot-Zn loading ability [27]. This also supports the speculation that ZIF1 could help Zn sequestration in the vacuole [27]. Therefore, the ZIF1 and its paralog ZIFL1 exhibit redundant functions for the Fe and Zn homeostasis. The rice seedlings exposed to excess Zn or Fe deficiency result in the differential changes of ZIFL genes at the transcriptional level [21]. Interestingly, Zn deficiency also regulates ZIFL genes expression in wheat [22].

Using expression studies and the loss-of-function approach, it was observed that plant ZIFL could also respond differentially to heavy metals stress (including nickel, Ni; and Cadmium, Cd). *Arabidopsis zif1* mutants have shown increased sensitivity to the presence of Cd, whereas a decreased sensitivity for the Ni was reported [19]. Similarly, mutants for *zifl2* show enhanced sensitivity to the exogenous levels of cobalt (Co), whereas, under excess copper (Cu) conditions, *zif1* mutants were sensitive [27]. Multiple wheat ZIFLs have been induced in the tissue-specific (roots and shoots) manner when exposed to metals such as Co, Cd and Ni [22]. This regulation has been speculated due to the heavy-metals responsive element (HMRE) [22]. Overall, these observations imply the role of ZIFL proteins during metal homeostasis. In addition to this, the transcriptional response of genes involved in micronutrient homeostasis is primarily due to the Fe-responsive cis-elements in the promoter sequences. The analysis of rice ZIFL promoters has revealed multiple binding sites for iron-deficiency responsive element-binding factor (IDEF1) transcription factor [21,22]. IDEF1 is a well-known ABI3/VP1 family of TFs involved in the Fe and Zn homeostasis. The presence of IDEF1 supports the regulation of plant ZIFL in Fe and Zn homeostasis [21].

### 2.2. Members as the Transporter of Mugineic Acids

**Figure 3:**
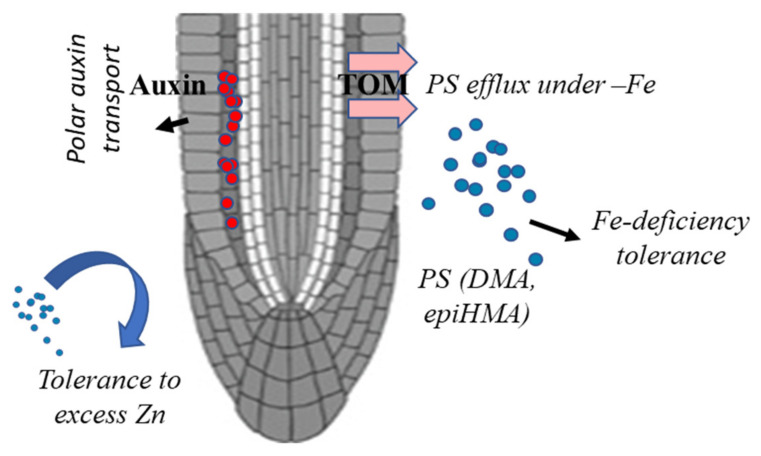
Root-specific functions of plant ZIFL transporters. The ZIFLs (TOMs) are involved in PS secretion in the rhizosphere to mobilize divalent cations with their extended function also in auxin homeostasis and tolerance to excess Zn. The colored round dots indicate auxins (red) and phytosiderophores (blue), such as deoxymugineic acid (DMA) and 3-epihydroxymugineic acid (epiHMA). The ZIFLs also provide tolerance to heavy metals (Ni, nickel; and Cd, cadmium) and excess zinc (Zn) condition.

Acquisition of Fe in the plant system is largely achieved by two different uptake strategies. Strategy I is the recruitment by dicots that involves the reduction and acidification activities of the rhizosphere. In contrast, most of the graminaceous plants rely mainly on strategy II processes in which chelators (including phytosiderophores, PS), such as mugineic acids (MAs), are secreted in the rhizosphere to form Fe-chelates [28]. The resulting Fe(III)-MA complex is taken up into maize and rice root cells by the YELLOW STRIPE 1 (YS1) and YELLOW STRIPE 1-like (*YSL*) transporters [29,30,31]. Interestingly, some of the plant ZIFLs have been characterized as the transporter of mugineic acid referred to as TOM [21,32]. In rice, three *TOM* genes were identified as proteins belonging to the ZIFL subfamily. Using C^14^-labeled deoxymugineic acid (DMA), the functionality of the rice ZIFL (transporter of mugineic acid1-TOM1) and barley was demonstrated in *Xenopus laevis* oocytes, thus proving them to be efflux transporters [32]. TOM1 shares 55% amino acid similarities with *AtZIFL1* family members. Overexpression of the TOM genes (*OsTOM1* and Barley TOM, *HvTOM1*) was able to mitigate the Fe deficiency symptoms, thereby providing clues for their ability to survive in the calcareous soils [32]. Subsequently, *OsTOM2* (ZILF5) was also characterized in rice for its role in internal transport of mugineic acid and metal homeostasis [33]. Using reverse genetics approaches, *TOM2* was shown to be involved in the translocation of Fe in shoots, and this is in contrast to the *TOM1* showing root-specific expression [32,33]. Using reporter assays, it was observed that both the OsTOM1 and OsTOM2 were found to be expressed in root stele. This suggested that DMA has the compensatory role as a chelator in Fe transport in xylem as a response to the Fe status of plant [32,33,34]. Besides Fe, DMA was shown to have the affinities for their ability to chelate Zn, Cu and Mn [35,36,37]. As represented in Figure 3, rice TOM transporters are able to secrete PS, including DMA and epiHMA, and therefore enable the mobilization of Fe and Zn to the roots. Crops, especially wheat and maize, when subjected to Fe deficiency stress, are capable of releasing a high amount of PS [38,39]. This, in turn, enables the higher mobilization of this micronutrient to the roots and helps in channelizing the Fe transport to the shoots via sym/apoplastic movement [40].

In hexaploid wheat, no functional evidence for the *TaZIFL* has been demonstrated to date. The differential expression of wheat ZIFL in response to Fe-deficiency condition co-relates with the efflux of PS [23]. Under Fe-deficiency, the release of PS was noted in several crop species [41,42,43]. Earlier, based on the sequence homology, three wheat TOMs, i.e., *TaTOM1*, *TaTOM2* and *TaTOM3*, were identified [22]. Interestingly, in maize it was demonstrated that under Fe-deficiency the release roots PS is affected by the diurnal pattern [44]. As shown in Figure 3, membrane-localized TOMs are the known effluxer of PS. This evidence indicates that ZIFL is a TOM protein that could eventually assist in the uptake and transport of Fe, two functions that are specific for cereal crops. Contrary to this, ZIF1 in *Arabidopsis* was characterized as a tonoplast-localized transporter and transporter of NA from the cytoplasm to the vacuoles [19,25]. Recently, the role of ZIFL has also been shown in grains, with their ability to alter the level of grain Fe and Zn [45]. In this study, a vacuolar localized ZIFL (Vacuolar Mugineic Acid Transporter; *OsZIFL12/VMT*) was identified that was highly expressed in node1 of the rice. The knockout lines in rice show enhanced accumulation of Fe and Zn by allowing the Fe(III)-DMA solubilization in the xylem sap, especially at the nodes [45]. Therefore, the role of manipulating the expression of the vacuolar localized ZIFL could be an exciting strategy to enhance both solubilization in the rhizosphere and the grain loading of the Fe(III). In addition, other functions of ZIFL, specifically in roots, have been reported (Figure 3), and those are discussed below.

## 3. ZIFL Could Provide Fe-Deficiency Tolerance

Genotype variation in the release of PS has been reported [46]. Earlier, the amount of the PS release was linked with the strength of the Fe-deficiency tolerance [47,48]. Monocot species have evolved mechanisms to tolerate conditions of low Fe availability by secreting PS that helps in Fe mobilization [35,46]. Under Fe-deficiency, the genes contributing to the PS biosynthesis are highly upregulated in roots [23]. Additionally, multiple genes, such as nicotianamine synthase, nicotianamine aminotransferase (NAAT) and 2′-deoxymugineic acid synthase, involved in PS biosynthesis are highly expressed in the roots; however, all the synthesized PSs may not be able to efflux in the rhizosphere [49,50,51]. Therefore, the high expression response of ZIFL genes is important for the PS release that has been linked to high Fe-translocation index from root to shoot. Additionally, since PSs have the ability to mobilize other cations, such as Zn, Mn and Cu, they have been shown to act as a ‘metallophores’ [52]. Cu and Zn deficiencies could also enhance PS release in different crops, such as maize, barley and wheat [53,54,55].

At the phenotypic level, Fe deficiency is marked with the chlorosis young leaves, as Fe is involved (co-factor) during chlorophyll biosynthesis. In fact, it has been observed that the shoot Fe status could determine the PS release in the roots, thereby showing the systemic response [56].They used biochemical and molecular studies to show that PS biosynthesis and release enzymes, such as NAAT and Ferritin, were not influenced by the Fe-deficiency in roots. In rice, tolerance to Fe deficiency is also dependent on the ferric chelate reductase (FRO) activity in the roots [57]. Rice could recruit both strategy-I- and II-mediated Fe-uptake pathways, whereas crops such as wheat, maize and barley are largely dependent on the latter mechanism [28]. Therefore, the tolerance to Fe-deficiency in these crops has been primarily attributed to their increased PS biosynthesis and release exudates (Figure 3). Using hormonal-inhibitory studies, the phenomenon of wheat tolerance to the Fe deficiency was linked to the partitioning of S-adenosyl methionine towards the ethylene and PS biosynthesis [58]. Large-scale screening of the wheat germplasm for its ability to secrete high PS was linked with efficient translocation of Fe in the shoots and eventually to the sink tissue, i.e., grain. Micronutrient biofortification has also been attempted to overexpress barley genes involved in PS biosynthesis in rice [49]. These transgenic lines show enhanced MAs production and also exhibit Fe-deficiency tolerance when grown in alkaline soils. This supports the notion of using TOM genes to ameliorate Fe-deficiency tolerance and micronutrient concentration in grains.

## 4. Antiporter Activity of ZIFL Is Linked to Auxin Homeostasis

The ZIFL proteins of the MFS possess antiporter activity that assist the sequential transport of two different molecules in opposite directions. This could suggest a broader ion transport role of ZIFL antiporters to help them survive in nutrient stress conditions. The antiporter activities are energy driven and are associated with the H+ motive force [59]. Many well-known antiporters are discussed elsewhere [60,61] that regulate a wide variety of physiological processes, including cell expansion, osmotic adjustment, pH regulation, membrane trafficking and cellular-stress responses. Some of the major known plant antiporters include Na-H^+^ exchanger (NHX), Ca^2+^/H^+^ exchangers (CAX) and K+ efflux antiporters (KEA) and are widely studied for their physiological roles [62]. The specific functions of the plant ZIFL, due to the presence of antiporter-conserved sequences, cause the ability to transport potassium (K^+^) and cesium (Cs^+^). Work in the model system, such as yeast, indicated that both the splice variants of *Arabidopsis AtZIFL* genes, referred to as AtZIFL1.1 and AtZIFL1.3, could facilitate H^+^-coupled K^+^ transport activity [63]. Their transportability in yeast mutants suggest no additional requirement of plant proteins, or it is possible that yeast has the necessary factor to support its function [63]. The ability of these ZIFL transporters to have antiporter activities supports the notion of their role in different physiological functions. Interestingly, AtZIFL1.1 was localized in the tonoplast and could indirectly help in auxin transport towards the shoot. In contrast, another splice variant, ZIFL1.3, as truncated protein, is localized at the plasma membrane of guard cells, thereby regulating stomatal functioning and providing drought tolerance [64]. Cell-to-cell transport of the auxin is primarily caused by the localization of the PIN auxin transporters. Experiments have pointed that the localization of auxin is, in fact, tightly regulated by the PIN transporters [65]. The transport of auxin in plants is linked with the plasma membrane H^+^(-ATPase) activity. Earlier, it was not known how the MFS transporters help in modulating polar auxin transport. In *Arabidopsis*, it was shown that auxin transport activity is indirectly dependent on ZIFL1 protein accumulation (Figure 3). Moreover, the AtZIF1 paralog, ZIF-like1 (ZIFL1), when expressed in yeast, induces the resistance to the synthetic 2,4-D via decreasing its concentration [64][. Therefore, it could be speculated that ZIFL1.1 is involved in the release of H^+^ from the vacuole that influences plasma membrane ATPases activity along with the proton-driving force to transport cellular auxin. Alternatively, it has also been hypothesized that ZIFL1.1 functions to normalize the plasma-membrane-localized PIN2 in epidermal root-tip cells when the roots are exposed to the conditions capable of triggering PIN2 degradation [64]. Overall, this work has demonstrated that ZIFL1.1 activity also enhances the PIN1 plasma membrane expression in the central cylinder cells [66]. This work indicated that ZIFL1.1 acts as a modulator of polar auxin transport in roots effectively, as represented in Figure 3.

In *Arabidopsis*, it has been reported that ZIFL2 is targeted to the plasma membrane of the endodermal and pericycle of root cells and is, therefore, necessary for K^+^ and Cs^+^ homeostasis [63]. Since Cs^+^ shows similar chemical properties to K^+^ ion, they compete for their transport. The activity of this *Arabidopsis* MFS carrier promotes cellular K+ efflux in the root, thereby restricting Cs^+^/K^+^ xylem loading and subsequent root-to-shoot translocation under conditions of Cs^+^ or high K^+^ external supply. Altogether, these studies demonstrate the upcoming varying role of the plant ZIFL, since it possesses antiporter activity, as well as the ability to secrete metal chelators, such as PS, in the rhizospheric regions (Figure 3).

## 5. Conclusions and Future Perspective

**Figure 4:**
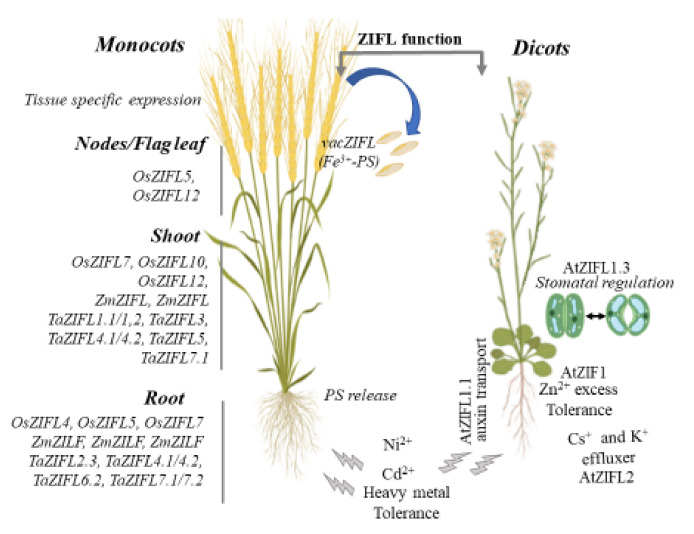
Overview of the plant ZIFL transporters characterized for their known functions. The list on the left side of the picture describes the tissue-specific expression response of the multiple ZIFL genes from crop plants, such as rice and wheat. On the right side of the image, ZIFL transporter functions are elaborated in the dicot, such as *Arabidopsis*. ZIFL’s function to provide tolerance to Ni and Cd stress seems to be conserved among the monocots and dicots.

The TOM family plays a pivotal role in the chelation, uptake and transport of Fe, mainly in cereal plants and hence is considered to be a potential target in enhancing micronutrient enrichment. Figure 4 reflects on how the plant ZIFL transporter, either from monocot or dicot, may operate for the diverse function and processes involving the efflux of MA/DMA, transport of K^+^ and regulation of auxin distribution. However, for now, these selected functions are restricted uniquely to the plant classes; therefore, investigations may be required for the expansion of these functions and new molecular players across the plant kingdom. The summarized tissue-specific expression of the crop plants’ ZIFLs and function studies in the diverse plant system suggest an upcoming role of this class of MFS subfamily proteins (Figure 4). Future studies will be directed towards identifying ZIFLs specific to new organelles, besides plasma membrane and vacuole. Moreover, their conserved and variable trans-membranous loop and the putative roles of these helical domains in probable protein–protein interactions akin to ABC transporters could be an area of future interest. This may reveal additional unidentified functions of MFS-ZIFL transporters. Employing the knock-in technology [67,68], using genome editing tools to introduce ZIFL with high-affinity transport and specificity for substrate, could help in expanding the horizons of these MFS subfamily functions.

**Table 1:** List of ZIFL homologs form the selected plant species. The table enlists a number of genes that have been identified and characterized for the function in the mentioned plant species (including *Zea mays*, *Oryza Sativa*, *Arabidopsis thaliana* and *Triticum aestivum*).

Name of the Plant Species	Gene Name	Characterized Function	References
*Zea mays*	*ZmZIFL1*, *ZIFL2*, *ZmZIFL3*, *ZmZIFL4*, *ZmZIFL5*, *ZmZIFL6*, *ZmZIFL7*, *ZmZIFL8*, *ZmZIFL9*, *ZmZIFL10*	ZIFL7: putative PS effluxer (TOM1)	[69,70]
*Oryza sativa*	*OsZIFL1*, *OsZIFL2 OsZIFL3*, *OsZIFL4 OsZIFL5*, *OsZIFL6 OsZIFL7*, *OsZIFL8 OsZIFL9*, *OsZIFL10 OsZIFL11*, *OsZIFL12 OsZIFL13*	ZIFL4: Transporter of mugineic acid in roots (TOM1)	[32]
		ZIFL5: cellular-transporter of mugineic acid/metal homeostasis (TOM2)	[33]
		ZIFL7: putative Transporter of mugineic acid (TOM3)	[33]
*Arabidopsis thaliana*	*AtZIF1*, *AtZIFL1*, *AtZIFL2*	ZIF1: Efflux transporter of NA	[25]
		ZIFL1: Zn tolerance	[19]
		ZIF1: Zn tolerance	[19]
		ZIFL 1.1: auxin homeostasis	[64]
		ZIFL 1.3: regulation of stomata	[64]
		ZIFL2: enhance metal tolerance	[71]
		ZIFL2: Cs and K homeostasis	[63]
*Triticum aestivum*	*TaZIFL1*, *TaZIFL2 TaZIFL3*, *TaZIFL4 TaZIFL5*, *TaZIFL6*, *TaZIFL7*	-	[23]
